# Animal Reproduction: Semen Quality Assessment

**DOI:** 10.3390/ani12212905

**Published:** 2022-10-23

**Authors:** Anna Wysokińska

**Affiliations:** Faculty of Agrobioengineering and Animal Husbandry, Siedlce University of Natural Sciences and Humanities, 08110 Siedlce, Poland; anna.wysokinska@uph.edu.pl; Tel.: +48-25-6431388

Semen quality is of fundamental importance for successful conception and embryonic development. Problems in animal reproduction have led to the search for more precise methods of semen assessment using ever newer techniques. Precise semen assessments are needed to predict male fertility and to optimize fertilization capacity in both natural conditions and assisted reproductive technology. Spermatozoa have a distinctive structure not found in other animal cells and exhibit high morphological variation. They are among the most diversified types of cells and are highly sensitive to various factors.

The purpose of the Special Issue ‘Animal Reproduction: Semen Quality Assessment’ is to present the latest scientific achievements in the use of advanced techniques for assessments of animal semen, including accurate analyses of sperm cell structures. Most of the studies published in the Special Issue are focused primarily on the effects of various factors on semen quality, e.g., the effect of antioxidants on the quality of cryopreserved semen and semen stored at room temperature; the impact of breed, testing method, and geographic location; the effect of semen storage time; and the influence of a semen extender. These studies, based on analyses of the semen of various animal species, including sheep, pigs, cattle, horses, and ostriches, touch on highly topical issues in animal reproduction.

Riesco et al. [1] carried out a multiparametric analysis of some previously tested antioxidants (crocin, GSH, and Trolox) and their effect on the quality of cryopreserved ram semen. The study showed beneficial effects of adding antioxidants to semen extenders for cryopreservation of sheep semen. For the first time, an analysis of semen quality in vitro was combined with an analysis of fertility following the artificial insemination procedure. The addition of 1 mM Trolox to a ram semen extender improved the quality of the thawed sperm, reducing cryodamage to the sperm and increasing fertility. The effectiveness of antioxidant treatments was tested for the first time in sheep, using an integrated and multiparametric approach combining in vivo and in vitro analyses and innovative approaches such as RedoxSYS. Cryopreservation causes various types of sperm damage. Therefore, the addition of antioxidants to semen extenders can be an effective tool for improving the cryopreservation of sheep semen and for optimizing artificial insemination.

Zhang et al. [2] conducted a study using various concentrations of taurine to test how this antioxidant affects the quality of semen stored at room temperature. This study was carried out on sheep of the Hu breed. The semen of this breed is highly sensitive to reactive oxygen species (ROS) during storage at room temperature. The semen was diluted with an extender supplemented with various concentrations of taurine (0, 10, 20, 40, 80, or 100 mM) at room temperature. The addition of taurine, especially at 20 mM, had a positive effect on semen quality relative to semen diluted using the extender without taurine (control). The sperm in the semen diluted using the extender with 20 mM of taurine showed better motility and cell membrane integrity as well as higher mitochondrial membrane potential compared to the control semen.

Perret et al. [3] investigated the effect of breed, testing method, and geographic location on the progressive motility of horse sperm. Semen quality expressed as sperm motility in horses is an important indicator of suitability for reproduction and fertility. The authors report that the breed of horse, testing methodology, and geographic location can significantly influence semen quality in horses and, thus, their reproductive potential. The assessment of progressive motility of the stallion sperm was based on an extensive review of data from 280 studies conducted in the years 1990–2018, followed by a meta-analysis. The temporal trends in sperm motility analysis indicate that the fertilization capacity of horse ejaculate has remained at a high level throughout the last three decades. Differences observed in the sperm motility parameters of stallions suggest that they are influenced by the method of assessment of this parameter, geographic differences, and individual variation among stallions. The authors emphasize the need for standardization of the methodology of sperm motility assessment in order to reduce variation resulting from testing techniques.

A study using the semen of Duroc x Pietrain crossbred boars and purebred boars of the parent breeds was conducted to evaluate changes in the cell membrane integrity of sperm taking place during semen storage [4]. Heterosis effects were estimated to compare the cell membrane integrity of the sperm heads of crossbred and purebred boars. The authors found that the cell membrane integrity of sperm heads changed with the passage of storage time for diluted semen but to different degrees in different breed groups. They noted that the semen of Duroc x Pietrain crossbreeds was less sensitive to storage conditions than that of boars of the parent breeds, which was confirmed by the heterosis effects. The semen of the crossbred boars contained a higher percentage of sperm with an intact cell membrane than in the case of purebred boars. Moreover, the semen of crossbred boars had significantly fewer dying sperm and sperm with a damaged cell membrane. The authors suggest that sperm cell membrane integrity should be evaluated more often during storage in the semen of Duroc and Pietrain boars than in the semen of Duroc x Pietrain crossbreeds.

An important research objective is the conservation of endangered animal species. This type of research was conducted by Jae-Wook Yoon et al. [5], who evaluated the semen quality of bulls of the Jeju Black Cattle (JBC) breed in order to select individuals with optimal reproductive traits. The authors evaluated bull semen in terms of sperm motility, viability, morphology, and total penetration rate. The analysis revealed that the semen of one of the individuals was of much higher quality than that of the others, which may suggest that this bull could be useful for the preservation and reproduction of endangered cattle breeds. Given the ageing population of JBC bulls, the authors suggest the need for a strategy to improve the quality of sperm produced in vivo.

Henning et al. [6] assessed semen preserved at 5 °C and stored for 24 h. The purpose of the research was to determine sperm traits of importance for fertility, i.e., the subpopulation structure of motile sperm and the ability of sperm to bind to oviduct explants in vitro, depending on the extender used and the storage time and temperature. Cooling boar semen to 5 °C and then storing it for 24 h at this temperature were shown to alter the subpopulation structure of motile sperm and to decrease the number of sperm capable of binding to the oviduct epithelium in vitro. The type of boar semen extender was also shown to affect changes in sperm induced by cooling to 5 °C.

Van der Horst and Maree [7] conducted an extensive review of research on the origin, migration, and reproduction of indigenous domestic animals, focusing on their semen quality. Their review was based on literature data and the authors’ own research on the semen quality of animals of several indigenous breeds. A comparison of several animal species and various indigenous and exotic breeds within these species revealed many similarities in semen parameters between native and exotic breeds. Based on their extensive analysis of their own data and literature data, the authors suggest that indigenous breeds are characterized by high semen quality and sperm functionality, similar to that of currently bred exotic breeds or crossbreds. The semen quality of these breeds is similar to that of current commercial breeds and has been quantified using the latest methods. In this context, the authors present functional tests of sperm enabling better estimation of semen quality than ordinary semen analyses.

Another study analysed the motility of ostrich sperm [8]. The analysis of ostrich semen is still at the experimental stage, and semen quality is not tested in this species before breeding. The aim of this study was to determine the relationship between sperm motility tested conventionally (using a phase-contrast microscope) and using computer-assisted sperm analysis (CASA). A positive relationship was shown between these methods with respect to sperm motility traits, which suggests that breeders could use the conventional, inexpensive method of motility assessment to test the quality of ostrich semen. However, the authors note the need to establish the correlation between these methods and fertility following artificial insemination or natural mating.

Frozen semen is rarely used in the artificial insemination of pigs due to its low quality after thawing. A study by Zhang et al. [9] investigated the effect of adding carboxylated ε-poly-L-lysine (CPLL) to semen extender as a cryoprotectant. The addition of 0.25% CPLL to the extender for freezing was found to significantly improve sperm motility, cell membrane integrity, mitochondrial function, and the antioxidant capacity of sperm after thawing. This study provides new information indicating that CPLL can be used as an effective cryoprotectant to improve the quality of cryopreserved boar semen after thawing.

The research presented in these published papers is very promising, provides a great deal of valuable knowledge, and can significantly contribute to advancements in animal reproduction. The editors thank all the researchers for their contributions to this Special Issue.

## Data Availability

No new data were created or analyzed in this study. Data sharing is not applicable to this article.
